# Antibiotic Susceptibility Testing (AST) Practices in India: Need for a National AST Forum

**DOI:** 10.7759/cureus.30971

**Published:** 2022-11-01

**Authors:** Kalyani Borde, Manick Dass, Ratna Mani Sharma, Dilip Mathai

**Affiliations:** 1 Microbiology, Apollo Institute of Medical Sciences and Research, Hyderabad, IND; 2 Microbiology, Apollo Hospitals, Hyderabad, IND; 3 Infectious Diseases, Apollo Institute of Medical Sciences and Research, Hyderabad, IND

**Keywords:** medical and diagnostic microbiology, antibiotic susceptibility testing, disc diffusion, india, quality control

## Abstract

Background

Accurate interpretation of antibiotic susceptibility testing (AST) is one of the most crucial functions of the microbiology laboratory. However, its performance depends on a number of critical factors. We conducted a status survey to understand the existing practices in Indian laboratories that have a potential to influence performance of AST.

Method

We developed a 22-point online survey questionnaire on information about respondent’s specifications, use of AST consumables, existing quality control protocols, and matters of contention in AST practices, and sent it by Google forms to 362 clinical microbiologists (holding MD or DNB certification). Participation was voluntary. Results were analyzed using descriptive statistics.

Results

Among 362, a total of 103 returned the questionnaire. The first 100 responses that were complete (all 22 questions answered) were analyzed. Respondents were from medical colleges (61%), private hospitals (26%), and stand-alone laboratories (13%). Analysis revealed that the Clinical & Laboratory Standards Institute (CLSI) guidelines were followed by all. Overall, 54% used disc diffusion as the primary method for performing AST. For the internal quality control testing of media and AST, 24% and 16% had adequate testing components and frequency, respectively. For performing AST of colistin, broth microdilution was used by 19%. Also, 86% participated in external quality control programs, and 54% respondents were dissatisfied or unsure about the development of competencies in AST methodology during their postgraduate training.

Conclusion

This survey reveals that potential gaps exist in the performance parameters and internal quality control of AST. There is an urgent need for harmonization in AST performance and postgraduate training in clinical microbiology in India.

## Introduction

Important in the armamentarium to tackle the growing threat of antimicrobial resistance (AMR) is its detection by antibiotic susceptibility testing (AST). The interpretative criteria for AST have evolved based on PK-PD parameters and emerging mechanisms of AMR. They have been carefully standardized by organizations such as Clinical & Laboratory Standards Institute (CLSI) and European Committee on Antimicrobial Susceptibility Testing (EUCAST) [1,2]. As these criteria are valid only under specific testing conditions, the onus lies with the laboratory performing AST to ensure that they are satisfactorily met. Quality control protocols have been defined to help laboratories produce reliable results [1,3]. Similarly, for external quality control (EQC) for AST results, the Indian Association of Medical Microbiologists (IAMM) program provides regular feedback to the participating laboratories [4,5].

Hence, we intended to conduct a survey to study the existing practices in AST performance in Indian laboratories by certified clinical microbiologists.

## Materials and methods

The study was approved by the institutional ethics committee (IRB/RC/2021/06/002). We designed a questionnaire to identify AST methodologies in microbiology laboratories. The survey included 22 questions covering important parameters regarding AST methodology as follows:

· Preliminary information about the respondents: (a) type of the laboratory, (b) place, (c) sample load, and (d) experience in years of the respondents.

· Consumables for AST: (a) availability of automation, (b) type of automated system for AST, (c) broth microdilution (BMD) or disk diffusion, (d) information about commercial/ in-house media, (e) type of media used for AST of fastidious organisms, (f) BMD for colistin susceptibility testing, and (g) disks used for performing AST.

· Quality control (QC) protocols: (a) components of internal QC testing, (b) QC of media, (c) QC of AST, (d) frequency of QC testing, (e) preparation of inoculum for AST testing, (f) participation in external quality assurance services (EQAS), and (g) guidelines followed for interpretation.

· Respondent’s opinion about matters of contention in AST methodology: (a) whether the microbiologist is consulted before purchasing AST supplies, (b) reason behind selection of AST supplies, (c) requests for AST for antibiotics lacking defined breakpoints, (d) clinical correlation of results, and (e) satisfaction with the training in AST methodology.

As the total number of practicing clinical microbiologists in India is unavailable, we used the directory of life members of IAMM, which enlists 3,600 members [6]. The sample size, accordingly, was calculated as 94 (confidence interval of 95% and margin of error at 10%).

Participation in the survey was voluntary. We shared this questionnaire on Google forms with the clinical microbiologists (n=362). We closed the survey within one month of sharing the questionnaire and analyzed the responses.

## Results

Out of 362, 103 (27%) agreed to participate in the survey. Of these 103, responses of the first 100 participants who had answered all the 22 questions were analyzed (Table 1).

**Table 1:** Important findings from the survey on antibiotic susceptibility practices in India. *More than one response per question was possible. AST, antibiotic susceptibility testing; EQAS, external quality assurance scheme; IQC, internal quality control; MHA, Meuller-Hinton agar; BMD, broth microdilution; DD, disk diffusion

Sl. no.	Parameter	Responses	%
1	Methodology for AST*	Disk diffusion	71
		Automated AST	36
2	Availability of automation for AST	Yes	63
		No	37
3	Name of the automated systems for AST	VITEK-2	88
		PHOENIX	11
		MicroScan WalkAway	1
4	Guidelines for interpretation	CLSI	100
		EUCAST	0
5	Participation in EQAS	Yes	86
		No	14
6	Components of IQC*	Sterility check of media	76
		Testing of control strains for specific antibiotics	68
		Thickness measurement of media	40
		pH of media	39
		None	5
7	Frequency of IQC for AST of control strains*	With each batch of media	53
		Once a week	28
		Once a month	20
		Once in three months	7
		Once in six months	3
		Never	12
8	Preparation of 0.5 McFarland inoculum	Use of densitometer	33
		Manual comparison with commercial standards	29
		Tentatively	20
		Manual comparison with in-house standards	18
9	Media for AST of fastidious organisms	5% sheep blood agar	44
		5% human blood agar	38
		MHA with 5% sheep blood	17
		Soybean casein agar with 5% sheep blood	1
10	BMD for colistin susceptibility	Yes	19
		No	81
11	Opinion of the microbiologist sought before purchasing consumables for AST	Always	68
		Sometimes	14
		Never	18
12	Factors affecting purchase decisions*	Internal validation	45
		Cost	34
		Trust and relations with the manufacturing company	17
		Troubleshooting support and subject expertise of manufacturer	4
13	Perception of clinical correlation of AST results	No major discrepancies perceived	47
		Unsure about clinical correlations due to lack of feedback	46
		Discrepancies observed often	5
		Discrepancies are never observed	3
14	Requests for testing and reporting of antibiotics without defined breakpoints	Yes	39
		Maybe	10
		No	51
15	Satisfaction with the post-graduate training regarding AST methodology	Yes	46
		Maybe	19
		No	35

Preliminary information

Respondents worked in the laboratory types as those attached to medical colleges (61%), private hospitals (26%), and stand-alone laboratories (13%). They were practicing in Karnataka (30), Telangana (23), Andhra Pradesh (13), Tamil Nadu (8), West Bengal (6), four each in Uttar Pradesh and Maharashtra, three each in Kerala and Odisha, two each in Assam and Delhi, and one each in Chhattisgarh and Gujarat. Sample load for culture/susceptibility testing was classified as high (>50 tests daily) for 32% laboratories, moderate (20-50 tests daily) for 47%, and low (<20 tests daily) in the remaining. Participants’ experience ranged from >10 years (38%), between 5 and 10 years (25%), and 1-5 years (37%), after obtaining the certification (MD/ DNB) in microbiology.

Consumables for AST

a. 71% used disc diffusion for AST, while 19% used only automated methods; 17% used a combination of both the methods.

b. The antibiotic discs were obtained from HiMedia laboratories by 87% and from more than one manufacturer by 20%.

c. 96% prepared media for AST in-house from dehydrated powders, and 87% obtained media from HiMedia laboratories.

d. 63% respondents had automation for AST.

e. Of those who had automation, 88% used VITEK®-2 system (bioMérieux, Marcy-l’Etoile, France), 11% used PHOENIX (BD, Sparks, MD), and 1% used MicroScan WalkAway (Dade Behring, Deerfield, IL).

f. For performing AST of fastidious organisms such as *Streptococcus*, 17% used Meuller-Hinton agar (MHA) with 5% sheep blood, while 38% used human blood agar (Table 1).

g. For colistin susceptibility testing, 19% performed BMD testing.

Quality control protocols

All the respondents used CLSI breakpoints for reporting AST, and 86% participated in EQC programs. Among the four components of internal quality control (IQC) testing of media (Table 1), 30% implemented any one, 26% implemented any two, and 39% implemented three or more components of IQC. No IQC protocols for AST media were implemented by 5% respondents.

IQC was performed with each new batch of media and/or discs by 53% of the respondents, and 16% performed IQC once a week and with each new batch of media and/or discs. No susceptibility testing was performed as a part of IQC by 12% respondents. The frequency of IQC was once a week for 28%, once a month for 20%, once in three months for 7%, and once in six months for 3% of the respondents.

Overall, 33% used a densitometer for preparing 0.5 McFarland of the inoculum, 20% adjusted the inoculum tentatively, while 18% and 29% adjusted the inoculum by manual comparison with in-house and commercial standards, respectively.

Respondent's opinions

Microbiologist’s opinions were always sought before purchasing consumables according to 68% of the respondents, and 18% and 14% were never or only sometimes consulted for the same, respectively.

Decisions based on quality of the products were made by 45% who also had a system in place for internal validation before making purchase decisions. However, for 34%, cost was the most important factor while selecting the AST consumables. Trust and relations with the manufacturing company were the most important factors affecting purchase decisions for 17%. Subject expertise and troubleshooting support provided by the manufacturer were the most important factors for 4%.

Out of all the respondents, 47% could not be sure whether the AST results correlated clinically due to lack of feedback from the clinicians. Also, 46% stated that there usually are no major discrepancies between laboratory results and clinical response, 5% observed discrepancies often, and 3% never observed such discrepancies.

Testing and reporting antibiotics with no defined breakpoints were requested to 39% of the respondents, while 10% stated that they were maybe approached for the same and 51% never faced this issue.

Of the respondents, 46% were satisfied with the training they received during their post-graduation period regarding AST methodology, 35% were dissatisfied, and 19% were unsure about it.

## Discussion

Reliability of AST depends on compliance with the carefully defined standards of performance. The interpretation of AST can vary widely when these standards are not met. This survey among practicing clinical microbiologists throws light on the status of AST performance in our region.

Medical college laboratories from the four states in southern India (n=79) are predominantly represented in this survey. Most respondents have >10 years of experience, closely followed by those with 1-5 years of experience.

One of the key findings from the survey is that the disk diffusion method for performing AST is most commonly used. This points toward the wide adaptation of disk diffusion methodology in India.

Disks as well as media were most commonly obtained from HiMedia laboratories, with other suppliers being Microexpress®, Oxoid™, and Bio-Rad laboratories. A study comparing the performance of antibiotic disks from various manufacturers found wide variations in the results when using same disks from different manufacturers [7]. Similarly, wide variation was observed in the performance of the dehydrated media from a few manufacturers [8]. No such study exists from India, severely restricting the ability of practicing microbiologists to make informed choices. A study from 2008, comparing three manufacturers, also highlighted the issues with variation of results [9]. A study from Nigeria demonstrated poor performance and variation in antibiotic content of locally manufactured disks as against imported disks [10]. Hence, it can be argued that there is a need to establish a reference laboratory for guiding practicing microbiologists in the country regarding the quality of commercially available supplies.

For automated AST, VITEK®-2 was the most commonly used instrument. Automated AST systems are increasingly being adapted by microbiologists in the country as they are easier to perform, are less labor-intensive, and provide quicker results. However, it is also important to note that most of these systems give extrapolated minimum inhibitory concentrations (MICs) and not the true MICs [11,12]. A national reference laboratory is needed to continuously monitor the antibiotic panels and performance of automated systems.

The main concern emerging from this survey is the lack of uniformly adapted quality control protocols for performing AST. Around half of the respondents performed inadequate components with infrequent IQC checks. A significant proportion of the respondents adjusted the inoculum tentatively or with crude methods. Also, only a minor proportion of respondents used appropriate media for performing AST of fastidious organisms, and a large proportion used routine blood agars. A disturbing reality is the extensive usage of human blood agar, which is not recommended by any methodology or performance standards.

All the respondents performed susceptibility testing of colistin, an important last-resort drug in the settings with high prevalence of carbapenem resistance. However, only a small proportion used the recommended method of BMD for the same, questioning the appropriateness of susceptibility results of this important antibiotic. Khurana et al. concluded in a single-center study from India that VITEK®-2 for colistin susceptibility testing is unreliable and suboptimal [13], necessitating wider adaptation of BMD.

A vast majority of the respondents opined that they are never or only sometimes consulted before making purchase decisions about the AST supplies. Also, less than half the respondents had a system of internal validation in place before making purchase decisions. This is an important area for improvement.

An indispensable role of the clinical microbiologist is correlating the AST results clinically. Almost half of the respondents could not comment about this due to lack of feedback from the clinicians. A case can be built to impart inter-professional communication skills among the clinical microbiologists as well as the clinicians if AST is to serve its purpose of guiding therapy.

In a survey by Anand et al., it was shown that more than 90% of the antibiotic FDCs (cefiderocol) were irrational [14]. There are no breakpoints available for these drugs. Along these lines, it is prudent for the clinical microbiologist to abstain from reporting such drugs. A significant proportion of the respondents agreed to being approached for testing and reporting such antibiotics for which breakpoints are not available. Under such circumstances, it is imperative that a national forum for AST decides which antibiotics can and cannot be reported in India.

It has become important to inculcate a clinical approach among the microbiologists in the country. More than half of the respondents were dissatisfied or unsure about the quality of training they received during their post-graduation studies. The experience in years of those who expressed dissatisfaction was almost evenly distributed between those with >10, 5-10, and 1-5 years. This again demonstrates a need to train young as well as older adults in this profession. As Wattal pointed out in 2018, the microbiology curriculum has not changed in the past 40 years, and also recommended major changes to train clinical microbiologists [15].

Limitations of the study include a probable biased sampling overrepresenting the microbiologists from the southern states and the government medical college establishments in the country. However, the public health and teaching hospital facilities are the ones needing immediate attention, given their dual role in reaching out to the vast underserved population of the country and training of future microbiologists.

Around 30 nations in the world, including China and South Africa, have constituted national AST committees in recent years. India, too, needs a guiding forum, which combines the expertise of microbiologists, infectious disease physicians, and pharmacologists, for harmonization of AST practices in this country. Its functions could be, but not limited to, those suggested in Table 2 [16].

**Table 2:** Suggested functions of a national forum for advising and training of the clinical microbiologists in the AST methodology. Adapted from EUCAST: suggestions on how to organize and form a NAC [16]. CLSI, Clinical & Laboratory Standards Institute; EUCAST, European Committee on Antimicrobial Susceptibility Testing

Suggestions	
1	To formulate susceptibility testing strategies at a national level.
2	To encourage quality assurance and to inform and support quality assurance agencies and accreditation programs.
3	To set up a website tailored to national needs in AST methodology
4	To provide education on AST methodology and interpretation.
5	To ensure adequate funding for AST methodology standardization.
6	To investigate and generate data to support breakpoints in comparison with CLSI/EUCAST in the Indian context.
7	To communicate with manufacturers of susceptibility testing material and devices.
8	To provide a technical representative for the international breakpoint committees.

## Conclusions

To summarize, major gaps exist in the IQC, supplies, and training in AST methodology in microbiology laboratories in our region. There is an urgent need to streamline the performance of inarguably the most important function in clinical microbiology laboratory - AST. A national AST committee could fill in the gaps by providing guidance and leadership to the microbiologists. Stakeholders and experts from multiple disciplines should undertake the difficult task of harmonization of AST performance in the country.
